# A CXCL10-Expressing Influenza Vector Induces Robust Adaptive Immunity Despite Strong Attenuation

**DOI:** 10.3390/pharmaceutics18060739

**Published:** 2026-06-14

**Authors:** Olga Ozhereleva, Alina Mustafaeva, Anastasia Pulkina, Marina Plotnikova, Marina Shuklina, Anna-Polina Shurygina, Marina Stukova, Andrej Egorov

**Affiliations:** Smorodintsev Research Institute of Influenza, The Ministry of Health of the Russian Federation, Saint Petersburg 197022, Russia

**Keywords:** influenza A virus, CXCL10, viral attenuation, T-cell response

## Abstract

**Background/Objectives:** Although influenza A viruses with partially truncated NS1 proteins are substantially attenuated and immunogenic due to enhanced innate immune activation; residual NS1-mediated antagonism of antiviral innate responses may support viral replication in the lower respiratory tract and constrain optimal immune responses. Strategies to further improve their immunogenicity and protective efficacy by incorporating immunomodulatory cytokines, such as IL-2, have been successfully explored. **Methods:** Here, we extended this approach to chemokine expression by engineering an NS1-truncated PR8-based virus (PR8/NS124) to express the immunomodulatory chemokine CXCL10 from the NS segment and compared it with the parental vector. **Results:** The recombinant NS124_SS_CXCL10 virus replicated to high titers in embryonated chicken eggs and MDCK cells. In vivo, however, CXCL10 expression reduced viral replication in mouse lungs by ~10^4^-fold, resulting in a near-non-replicating phenotype. In contrast to the parental virus, the vector did not induce weight loss and exhibited a strongly attenuated phenotype. This effect was associated with altered innate immune signaling, including increased IRF7 expression and early induction of IFN-α responses in the lungs, together with modulation of TLR-dependent sensing pathways in the upper respiratory tract. Despite severely impaired replication, intranasal immunization induced antigen-specific T-cell responses comparable to those elicited by the parental vector. Following intraperitoneal immunization, when replication of both vectors was minimal, the CXCL10-expressing vector induced significantly higher frequencies of antigen-specific CD8^+^ and CD4^+^ effector-memory T cells. This was accompanied by enhanced antigen-specific T-cell recall responses in the lungs following intranasal challenge. Importantly, the CXCL10-expressing vector demonstrated protective efficacy comparable to that of the parental NS124 vector against heterologous H3N2 challenge while exhibiting an improved safety profile. **Conclusions:** These findings support the incorporation of CXCL10 as a strategy to improve the safety and T-cell immunogenicity of NS1-truncated influenza vectors.

## 1. Introduction

Viruses can modulate host immune responses to support their replication, in part by suppressing cytokine and chemokine production. Influenza A virus achieves this immune evasion primarily through non-structural protein 1 (NS1), a key virulence factor that antagonizes host innate and adaptive immune responses by inhibiting type I interferon induction, antiviral signaling, dendritic-cell activation, and the development of effective T-cell immunity [1,2,3,4]. Accordingly, truncation or deletion of NS1 leads to virus attenuation while enhancing innate immune stimulation.

Structurally, NS1 contains several functional domains that enable its immune evasion activity. The N-terminal RNA-binding domain (RBD) interferes with antiviral sensing pathways, whereas the C-terminal effector domain (ED) mediates interactions with host proteins involved in immune regulation and mRNA processing [4,5,6].

The degree of attenuation caused by NS1 truncation correlates with disruption of its immune evasion functions [7]. Complete deletion of NS1 results in replication-deficient viruses that fail to propagate in interferon-competent systems and cannot be efficiently amplified in MDCK cells or embryonated eggs [8]. In contrast, partial truncation preserves limited immune evasion while enhancing innate immune activation, and supports productive replication in manufacturing substrates and the respiratory tract, albeit with a significantly attenuated phenotype [7].

The NS1-truncated mutant retaining the first 124 amino acids (NS124) represents a platform that balances attenuation with replication competence: it preserves the RNA-binding domain but lacks most of the effector domain. This enables virus propagation to high titers in embryonated eggs while maintaining an attenuated phenotype [7,9,10]. However, NS124 retains the ability to replicate in the respiratory tract, including in the lungs, and at high doses, can induce pathology. Moreover, preservation of the RNA-binding domain allows for partial suppression of viral RNA sensing and downstream innate pathways. NS1 proteins with an intact N-terminal domain can inhibit caspase-1 activation and limit the maturation of interleukin-1β (IL-1β) and IL-18, key mediators of inflammasome-dependent signaling [11]. As these cytokines contribute to effective adaptive immune responses, this residual immune evasion may restrict the full immunostimulatory potential of NS124. Thus, further optimization of both safety and immune activation remains an important objective.

Incorporation of immunomodulatory sequences into the viral genome represents a strategy to enhance the immunogenicity of NS1-modified influenza vectors. Expression of cytokines such as interleukin-2 (IL-2) from the NS segment has been shown to enhance antiviral immune responses, supporting the concept of NS1-modified viruses as platforms for immune modulation [1,12].

Chemokines can complement this strategy by directing the recruitment and spatial organization of immune cells. CXCL10 is of particular interest due to its central role in antiviral immunity, as it interacts with the CXCR3 receptor expressed on activated CD8^+^ T cells, NK cells, and Th1-polarized CD4^+^ lymphocytes [13,14,15]. This axis promotes effector-cell migration to sites of infection, supports Th1 polarization, and enhances cellular immune responses. In addition to its chemotactic function, CXCL10 can modulate interferon-dependent signaling pathways, thereby amplifying antiviral immunity [16,17,18].

In the present study, we generated an NS124-based influenza A virus vector expressing CXCL10 and evaluated its replication, attenuation, and immunogenicity. We further investigated its effects on innate immune activation, T-cell responses, and protective efficacy in comparison to the parental NS124 vector.

## 2. Materials and Methods

### 2.1. Cells

Vero (ATCC #CCL-81, Manassas, VA, USA) and MDCK (#FR-58; IRR, Manassas, VA, USA) cell lines were used. Vero cells were cultivated in OptiPro medium (Gibco, Grand Island, NY, USA) with 2% GlutaMax (Gibco, Grand Island, NY, USA). MDCK cells were cultured in AlphaMEM medium (Biolot, Saint Petersburg, Russia) supplemented with 10% SC-biol fetal serum (Biolot, Saint Petersburg, Russia).

### 2.2. Viruses

The recombinant PR8/NS124 strain, generated by reverse genetics and encoding an NS1 protein truncated to 124 amino acids as previously described [7], was used as the control parental vector. A/Puerto Rico/8/1934 (H1N1) wild-type virus was used for low-dose mouse challenge experiments and for in vitro stimulation assays to assess adaptive immune responses. A mouse-adapted A/Aichi/2/68 (H3N2) strain was used for heterologous challenge studies. All viral stocks were propagated in embryonated chicken eggs (ECEs).

### 2.3. Animals

Female C57Bl/6 mice (16–18 g) were acquired from the Laboratory Animal Nursery Pushchino (Shemyakin and Ovchinnikov Institute of Bioorganic Chemistry RAS, Moscow, Russia) or from the “RAPPOLOVO” laboratory animals nursery state facility of the Russian Academy of Medical Sciences. All procedures involving animals were performed in compliance with international regulations (Directive 2010/63/EU) and were approved by the local Bioethics Committee of the Smorodintsev Research Institute of Influenza.

### 2.4. Generation of Recombinant Virus

Recombinant influenza virus A/PR8/NS124-CXCL10 (H1N1) based on the A/PR/8/1934 strain with the NS124-CXCL10 chimeric protein obtained by reverse genetics [19]. Vero cells were transfected with a set of 8 bidirectional plasmids encoding influenza virus genes using the Nucleofector II (Amaxa, Cologne, Germany) and the Nucleofection Kit V reagent kits (Lonza #VCA-1003, Basel, Switzerland). After transfection, cells were incubated in medium containing 1 μg/mL TPCK-trypsin (Sigma-Aldrich, St. Louis, MO, USA) until a specific cytopathic effect developed, and then propagated in 10–12-day-old ECEs (Sinyavinskaya Poultry Farm, Sinyavino, Russia).

The infectious activity was assessed by the limiting dilution assay in MDCK cells and ECEs. Virus dilutions for infection of MDCK cells were prepared in α MEM medium containing 1% antibiotic-antimycotic (Gibco, Grand Island, NY, USA) and 1 μg/mL TPCK-trypsin. Dilutions for infection of ECEs were prepared in DPBS buffer (Biolot, Saint Petersburg, Russia) with 1% antibiotic-antimycotic. The 50% tissue culture infectious dose (TCID_50_) and egg infectious dose (EID_50_) were calculated according to the Reed and Muench method and expressed as log_10_ values [20].

### 2.5. CXCL10 Quantification

Chemokine expression was quantified in allantoic fluid by ELISA. Ten-day-old ECEs were infected with recombinant CXCL10 vector (2 × 10^5^ PFU/egg), and allantoic fluid was collected 48 h post-infection. CXCL10 concentrations were measured using LEGEND MAX™ Human CXCL10 (IP-10) ELISA kits (BioLegend, San Diego, CA, USA) according to the manufacturer’s instructions.

### 2.6. Immunization

A total of four independent animal experiments were conducted in this study. To minimize experimental variability, all viral vectors were amplified once in embryonated chicken eggs, aliquoted, and stored at −70 °C. For each experiment, aliquots from the same virus stock were thawed and diluted immediately before administration to obtain the required immunization or challenge doses. Thus, all in vivo studies were performed using virus preparations derived from the same original stock. All mice were female C57BL/6 animals that were age-matched, obtained from the same supplier, and maintained under identical housing conditions throughout the study.

Experiment 1 evaluated viral replication, innate immune responses, and protective efficacy following intranasal immunization with 6 log_10_ EID_50_ per mouse in a total volume of 30 μL. Antiviral gene expression was assessed in lungs and nasal turbinates at 8 and 24 h post-immunization, and lung viral titers were determined on day 3. Protective efficacy was evaluated 21 days later following heterologous challenge with A/Aichi/2/68 (H3N2) at a dose of 6 log_10_ EID_50_ in a total volume of 10 μL per mouse.

Experiment 2 assessed safety and adaptive immune responses following intranasal immunization with 7 log_10_ EID_50_ per mouse in a total volume of 30 μL. Body weight was monitored and antigen-specific T-cell responses in lungs and spleens were analyzed 14 days post-immunization.

Experiment 3 examined innate and adaptive immune responses following intraperitoneal immunization with 7 log_10_ EID_50_ per mouse. Peritoneal lavage samples were collected at 12 and 24 h post-immunization for analysis of innate immune cell recruitment and cytokine production. Adaptive immune responses were assessed in the spleen 14 days after immunization. Recall responses were evaluated following low-dose intranasal PR8 challenge at a dose of 3 log_10_ EID_50_ in a total volume of 30 μL per mouse.

Experiment 4 quantified local type I interferon responses after intranasal immunization with 6 log_10_ EID_50_ per mouse in a total volume of 30 μL by measuring IFN-α concentrations in bronchoalveolar lavage samples collected at 8 and 24 h post-immunization.

Detailed experimental timelines, sampling schedules, and challenge procedures are provided in Supplementary Figure S1.

### 2.7. Evaluation of Viral Infectious Activity

Virus shedding was assessed on days 3 and 5 post-immunization. Mice were euthanized, and their lungs and nasal turbinates were harvested and homogenized using a TissueLyser II bead homogenizer (Qiagen, Hilden, Germany). Virus titers were measured by infecting MDCK cells with tissue homogenates and subsequently analyzing hemagglutination assays using a suspension of chicken red blood cells (0.5%). Tissue infectivity was quantified as the 50% tissue culture infectious dose (TCID_50_), calculated with the Reed and Muench method [20], and expressed as log_10_ TCID_50_/mL.

### 2.8. PCR Analysis

Quantitative real-time PCR (qPCR) was performed using a GenTier 96E PCR Cycler (TianLong, Xi’an, China). Multiplex reactions were carried out in a final volume of 25 µL, which contained 12.5 µL of 2× BioMaster HS-qPCR mix (BioLabMix, Novosibirsk, Russia), 5 µL of cDNA template, 300 nM of each primer, and 200 nM of the corresponding TaqMan probe. Amplification was performed under the following cycling conditions: initial denaturation at 95 °C for 5 min, followed by 40 cycles of 95 °C for 10 s, 58 °C for 10 s, and 72 °C for 10 s [21]. Primer and probe sequences used for gene expression analysis are provided in Supplementary Table S1 (mouse tissues).

For mouse samples, Ct values were normalized to the geometric mean of multiple housekeeping genes (Hprt1, GAPDH, Ubc, and Rplp0), whereas GAPDH was used as the reference gene for human cell samples. Relative gene expression was calculated using the comparative ΔΔCt method with untreated biological controls as calibrators. Fold changes were determined according to the formula 2^−ΔΔCt^.

### 2.9. IFN-α Quantification

BAL samples were collected 8 and 24 h post-immunization by flushing the airways with 500 μL of sterile PBS. IFN-α concentrations were measured using the Mouse IFN Alpha ELISA Kit (Invitrogen, Carlsbad, CA, USA) according to the manufacturer’s instructions.

### 2.10. Cytokine Production Analysis

Cytokine concentrations in peritoneal washes were determined using the LEGENDplex multiplex system (Biolegend, San Diego, CA, USA) according to the manufacturer’s instructions.

### 2.11. Leukocytes Isolation and Stimulation

Briefly, leukocytes isolated from the lungs, spleen, and peritoneal washes were harvested from mice [22] and mechanically dissociated. Lung tissue was additionally digested with collagenase/DNase (Sigma, Saint Louis, MO, USA). Cells were filtered through 70 µm strainers, and erythrocytes were lysed using RBC lysis buffer (BioLegend, San Diego, CA, USA). Prepared single-cell suspensions were seeded at a density of 1 × 10^6^ cells per well in 96-well plates (Nunc, Roskilde, Denmark). For intracellular cytokine staining (ICS), cells were stimulated with 5 µg/mL influenza A/PR8 virus or with the NP_366–374_ peptide (Verta Ltd., Saint Petersburg, Russia) in the presence of brefeldin A (BioLegend, San Diego, CA, USA).

### 2.12. Flow Cytometry Analysis

To identify cytokine-producing CD4+/CD8+ Tem cells, samples were stained with CD8-PC7, CD4-PC5.5, CD62L-APC-A750, CD44-KO525, IFNγ-FITC, TNFα-PB450, and IL2-PE antibodies (BioLegend, San Diego, CA, USA) using the Solution for Fixation and Permeabilization (BD Biosciences, San Jose, CA, USA). Phenotyping of innate immune cells was performed using a panel of fluorophore-conjugated antibodies: CD11b-PE/Cy7, CD11c-PE, MHCII-Alexa Fluor 488, Ly6G-PerCP-Cy5.5, Ly6C-Alexa Fluor 700, CD103-BV605, CD45-APC/Cy7, CD64-BV421, and CD24-BV510 (all from BioLegend, San Diego, CA, USA). The following cell populations were identified based on established surface marker expression: neutrophils (SSChiCD45+Ly6G+), macrophages (CD45+MHCII+CD64+CD11c/CD11b+), monocytes (CD45+MHCII-CD64+CD11c/CD11b+), and dendritic cells (CD45+MHCII+CD64-CD24+CD11c/CD11b+CD103+/CD103-). Data were collected using a CytoFlex flow cytometer (Beckman Coulter, Bray, CA, USA) and analyzed with Kaluza Analysis software (v2.2) (Beckman Coulter, Bray, CA, USA).

### 2.13. Statistical Analysis

Data analysis was conducted using GraphPad Prism 10.0 (GraphPad Software, Inc., San Diego, CA, USA). Results are presented as mean ± standard deviation (SD) or standard error of the mean (SEM). Statistical comparisons were performed using one-way or two-way ANOVA or an unpaired *t*-test.

## 3. Results

### 3.1. Efficient Rescue of a Recombinant Influenza Vector Expressing CXCL10

To generate influenza vectors expressing CXCL10, we used the A/Puerto Rico/8/34 (H1N1) strain (PR8) carrying a truncated NS1 protein of 124 amino acids (PR8/NS124). To enable chemokine expression, we employed the overlapping stop/start codon strategy (TAATG) described by Kittel et al. for expressing human IL-2 [3]. Briefly, the upstream stop codon terminated translation of the NS1 protein at 124 amino acid residues, while the downstream AUG initiated translation of the chemokine open reading frame, including its native signal peptide.

Recombinant influenza viruses were rescued by plasmid transfection of Vero and MDCK cells and subsequently propagated in 10-day-old ECEs. The CXCL10-expressing vector was successfully propagated in the allantoic cavity, reaching hemagglutination titers of 1:64.

**Figure 1:**
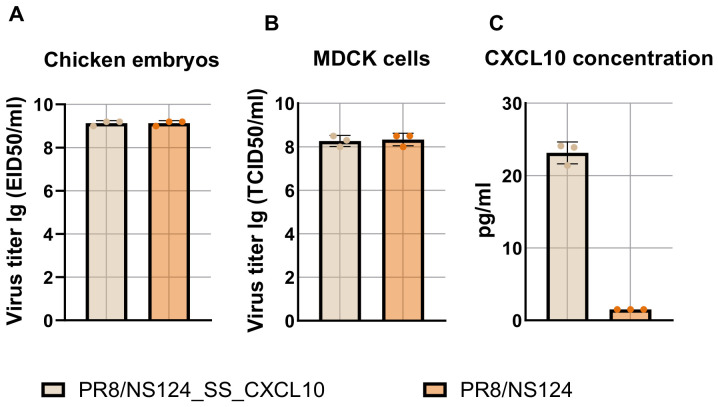
Replication of Recombinant Influenza Vectors and CXCL10 Expression. Replication of the NS124_SS_CXCL10 virus was evaluated in ECEs (**A**) and MDCK cells (**B**). To assess CXCL10 production (**C**), 10-day-old ECEs were infected with NS124_SS_CXCL10, and allantoic fluid was collected 48 h post-infection. CXCL10 concentrations were measured using the LEGEND MAX™ Human CXCL10 (IP-10) ELISA Kit.

The NS124_SS_CXCL10 vector replicated efficiently in ECEs and MDCK cells, reaching titers of up to 9.0 and 8.0 log_10_, respectively, comparable to those of the parental PR8/NS124 virus (empty vector control; Figure 1).

Chemokine expression in the allantoic fluid was quantified by ELISA. ECEs were infected with 2 × 10^5^ PFU of the CXCL10 vector per egg. Allantoic fluids were harvested 48 h post-infection and analyzed using the LEGEND MAX™ Human CXCL10 (IP-10) ELISA kits (BioLegend, USA). The results demonstrated production of CXCL10 (23.5 ± 3.7 pg/mL; Figure 1), confirming efficient replication and transgene expression.

### 3.2. CXCL10 Expression Limits Viral Replication and Prevents Weight Loss in Mice

To assess the safety profile of the CXCL10-expressing vector, C57BL/6 mice were intranasally immunized with a high dose (7 log_10_ EID_50_) of NS124_SS_CXCL10 or the parental NS124 virus. Body weight dynamics were monitored as a readout of reactogenicity.

**Figure 2:**
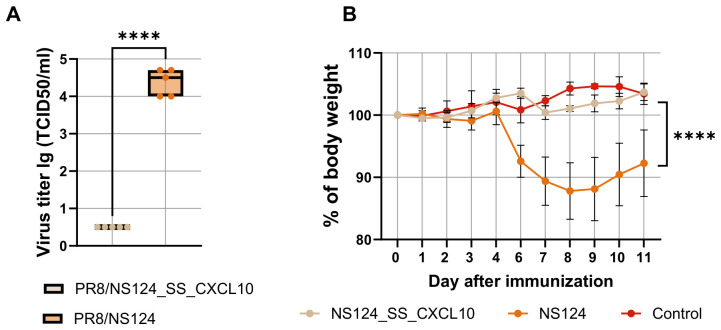
Viral Load and Body Weight Following Intranasal Immunization with the NS124_SS_CXCL10 Influenza Vector. Viral load in the lungs (**A**) was assessed in C57BL/6 mice immunized intranasally at a dose of 6 log_10_ EID_50_/30 μL (*n* = 5). Lung tissues were collected on day 3 post-immunization, and viral titers were determined by titration of tissue homogenates in MDCK cells. Body weight dynamics following intranasal immunization are shown in (**B**). C57BL/6 mice were immunized intranasally with 30 μL of 7 log_10_ EID_50_ per mouse (*n* = 10); control animals received PBS. Data are presented as mean ± SD. Statistical significance was determined using an unpaired *t*-test (**A**) and two-way ANOVA followed by Tukey’s multiple comparison test (**B**) (**** *p* < 0.0001).

High-dose intranasal immunization revealed clear differences in tolerability between the vectors. Mice immunized with the parental NS124 virus exhibited pronounced weight loss, reaching approximately 10–12% by days 7–9 post-immunization. In contrast, animals administered the CXCL10-expressing vector maintained stable body weight comparable to control animals, indicating reduced pathogenic effects despite the high dose used (Figure 2).

In addition, following intranasal immunization of C57BL/6 mice with 6 log_10_ EID_50_ per mouse, the NS124_SS_CXCL10 vector exhibited a high degree of attenuation, with an almost replication-defective phenotype. In contrast, the parental PR8/NS124 virus replicated in the lungs, reaching titers of 4.5 ± 0.4 log_10_ (Figure 2). While both vectors replicated efficiently in ECEs and MDCK cells, which lack a complete immune response, replication of the CXCL10-expressing vector in mice was markedly reduced, suggesting enhanced immune-mediated restriction in vivo.

### 3.3. CXCL10 Differentially Modulates Innate Immunity and Interferon Signaling in the Lungs and Nasal Turbinates

To investigate the mechanisms underlying attenuation of the NS124_SS_CXCL10 vector, we analyzed the expression of innate immune response genes in the nasal turbinates and lungs of C57BL/6 mice at 8 and 24 h post-immunization (30 μL, 6 log_10_ ED_50_ per mouse).

**Figure 3:**
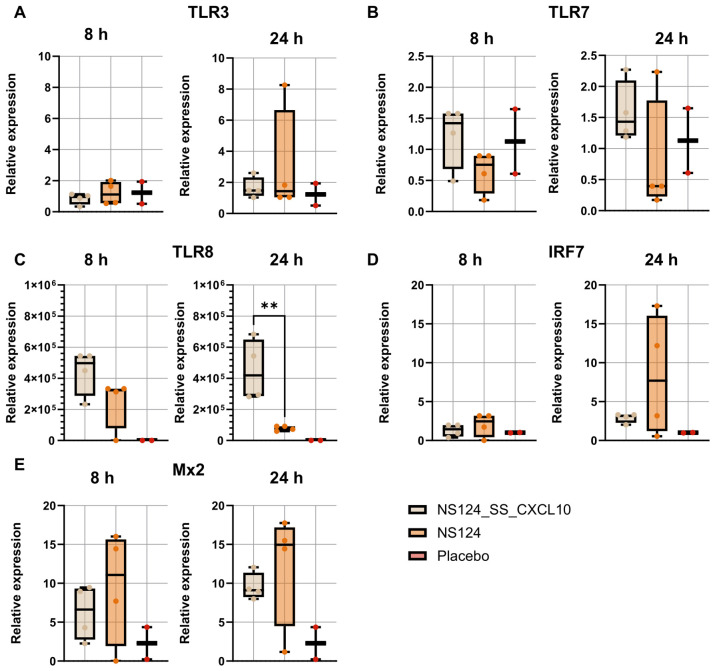
Relative mRNA expression levels of TLR3, TLR7, TLR8, TLR9, IRF7, and Mx2 in mouse nasal turbinates 8 and 24 h after immunization with NS124 and CXCL10-expressing vector (NS124_SS_CXCL10). C57BL/6 mice were immunized intranasally with the NS124_SS_CXCL10 (*n* = 4) or the NS124 (*n* = 4) strains at a dose of 6 log_10_ EID_50_ per mouse. The control group (*n* = 2) received an equivalent volume of PBS (30 μL per mouse). Lungs were collected 8 and 24 h after immunization. mRNA levels were measured using Real-time PCR. Relative expression levels of TLR3 (**A**), TLR7 (**B**), TLR8 (**C**), IRF7 (**D**), and Mx2 (**E**) are presented as box-and-whisker plots (minimum to maximum, with individual values and the median indicated). The control group (*n* = 2) was only used to establish the baseline level of gene expression for ΔΔCt calculations and was not included in statistical comparisons. Statistical comparisons were only performed between NS124_SS_CXCL10 and NS124 groups (*n* = 4 per group) using an unpaired *t*-test (**: *p* < 0.01).

In the nasal turbinates, expression levels of most analyzed genes were comparable between the NS124_SS_CXCL10 and NS124 groups. However, the CXCL10-expressing vector induced significantly higher TLR8 expression at 24 h post-immunization and showed a tendency toward increased TLR7 expression, suggesting enhanced recruitment of innate immune cells to the primary site of infection (Figure 3).

**Figure 4:**
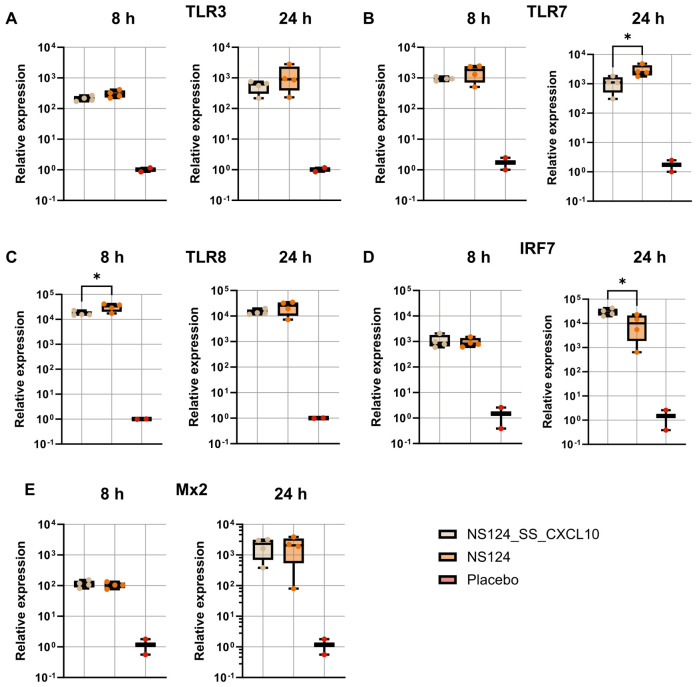
Relative mRNA expression of TLR3, TLR7, TLR8, TLR9, IRF7, and Mx2 in mouse lungs 8 and 24 h after immunization with the parental NS124 vector and CXCL10-expressing vector (NS124_SS_CXCL10). C57BL/6 mice were immunized intranasally with the NS124_SS_CXCL10 (*n* = 4) or the NS124 (*n* = 4) strains at a dose of 6 log_10_ EID_50_ per mouse. The control group (*n* = 2) received an equivalent volume of PBS (30 μL per mouse). Lungs were collected 8 and 24 h after immunization. mRNA levels were measured using Real-time PCR. Relative expression levels of TLR3 (**A**), TLR7 (**B**), TLR8 (**C**), IRF7 (**D**), and Mx2 (**E**) are presented as box-and-whisker plots (minimum to maximum, with individual values and the median indicated). The control group (*n* = 2) was only used to establish the baseline level of gene expression for ΔΔCt calculations and was not included in statistical comparisons. Statistical comparisons were only performed between NS124_SS_CXCL10 and NS124 groups (*n* = 4 per group) using an unpaired *t*-test (* *p* < 0.05).

In the lungs, distinct patterns of innate immune activation were observed. The parental NS124 vector was associated with higher expression of TLR7 and TLR8, whereas the CXCL10-expressing vector induced significantly higher IRF7 expression at 24 h post-immunization. These findings suggest that CXCL10 expression promotes activation of interferon-associated signaling pathways despite the absence of enhanced viral sensing receptor expression (Figure 4).

**Figure 5:**
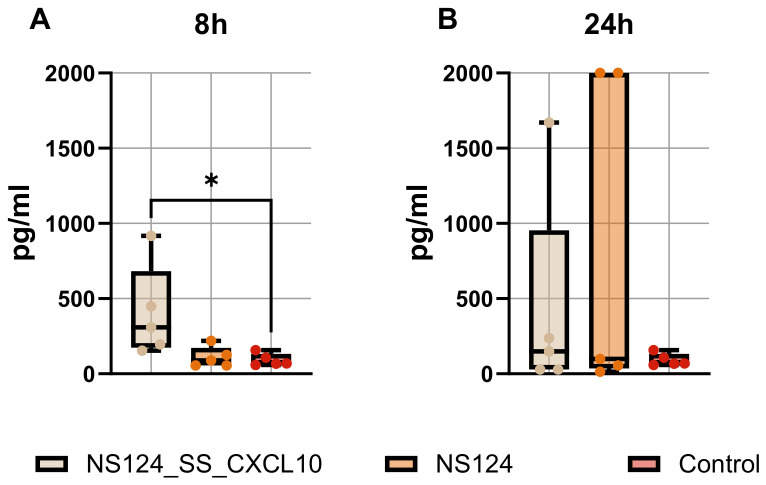
IFN-α production in bronchoalveolar lavage at 8 and 24 h after immunization. C57BL/6 mice were immunized intranasally with the NS124_SS_CXCL10 (*n* = 5) or the NS124 (*n* = 5) strains at a dose of 6 log_10_ EID_50_ per mouse. The control group (*n* = 5) received an equivalent volume of PBS (30 μL per mouse). BAL samples were collected at 8 h (**A**) and 24 h (**B**) post-immunization. IFN-α concentrations were determined by ELISA and are presented as box-and-whisker plots (minimum to maximum, with individual values and the median indicated). Statistical significance was assessed by one-way ANOVA followed by Tukey’s multiple comparison test (* *p* < 0.05).

To determine the activation of type I interferon responses, IFN-α levels were measured in BAL. At 8 h, mice immunized with the NS124_SS_CXCL10 vector exhibited significantly higher IFN-α concentrations compared with the control group, indicating a more rapid induction of antiviral signaling (Figure 5). By 24 h, IFN-α levels increased in the NS124 group, likely reflecting ongoing viral replication and sustained innate immune activation.

To further characterize innate immune modulation beyond transcriptional profiling, we also evaluated innate immune cell populations and cytokine responses following immunization. No major differences between the CXCL10-expressing and parental vector were observed in innate immune cell composition or early cytokine production. This suggests that CXCL10-mediated attenuation does not involve broad enhancement of inflammatory cell recruitment or cytokine induction. The relevant data are presented in the Supplementary Materials (Figures S2 and S3).

Overall, these findings indicate that although both vectors can activate innate immunity, CXCL10 expression shifts the response from enhanced viral recognition toward amplification of interferon-dependent signaling pathways.

### 3.4. Adaptive Immune Responses Following Intranasal Immunization Are Preserved Despite CXCL10-Mediated Attenuation

To determine whether CXCL10 expression preserves adaptive immunity under attenuated conditions, we evaluated T-cell responses following intranasal immunization (7 log_10_ EID_50_) with NS124_SS_CXCL10 or the parental NS124 vector in C57BL/6 mice.

**Figure 6:**
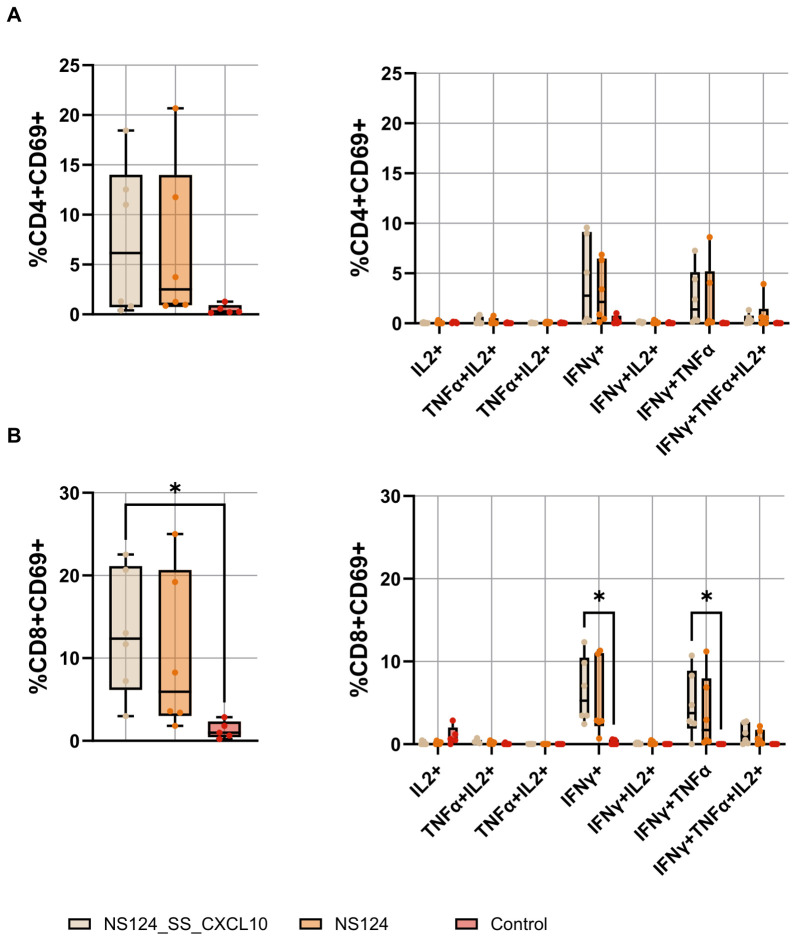
Antigen-specific CD4^+^ and CD8^+^ tissue-resident memory T-cell (Trm) responses in the lungs. To measure Trm responses in the lungs, C57BL/6 mice were immunized intranasally with NS124_SS_CXCL10 (*n* = 6) or NS124 (*n* = 6) at a dose of 7 log_10_ EID_50_ per mouse. The control group (*n* = 5) received an equivalent volume of PBS (30 μL per mouse). Lungs were collected at 14 d.p.im. Trm response in the lungs was evaluated by intracellular cytokine staining after 6 h of in vitro stimulation with the NP366–374 peptide. The total percentage of cytokine-producing CD4+ Trm cells and the percentage of CD4+ Trm cells producing any combination of IFN-γ, IL-2, or TNF-α (**A**) and the total percentage of cytokine-producing CD8+ Trm cells and the percentage of CD8+ Trm cells producing any combination of IFN-γ, IL-2, or TNF-α (**B**) are presented as box-and-whisker plots (minimum to maximum), with individual values and the median indicated. Data were considered statistically significant at *p* < 0.05, as determined by one-way or two-way ANOVA followed by Tukey’s multiple comparison test (*: *p* < 0.05).

**Figure 7:**
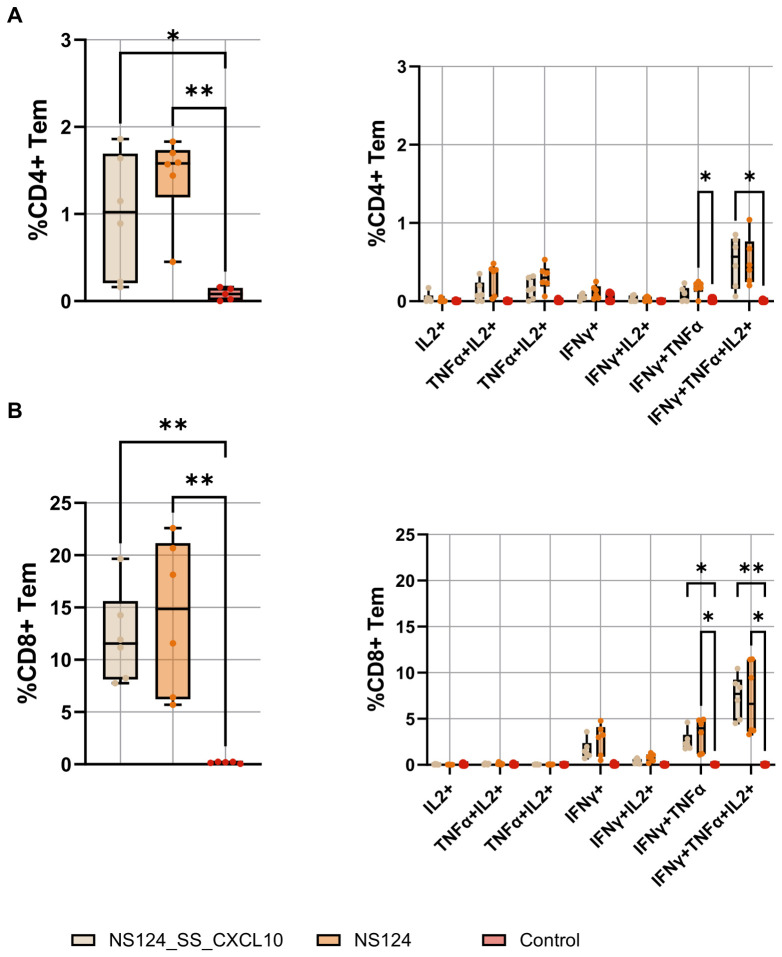
Antigen-specific CD8+ and CD4+ effector-memory T cells (Tem) responses in the spleen. To measure Tem responses in the spleen, C57BL/6 mice were immunized intranasally with NS124_SS_CXCL10 (*n* = 6) or NS124 (*n* = 6) at a dose of 7 log_10_ EID_50_ per mouse. The control group (*n* = 5) received an equivalent volume of PBS (30 μL per mouse). Spleens were collected at 14 d.p.im. Tem response in the spleens was evaluated by intracellular cytokine staining after 6 h of in vitro stimulation with the NP366–374 peptide. The total percentage of cytokine-producing CD4+ Tem cells and the percentage of CD4+ Tem cells producing any combination of IFN-γ, IL-2, or TNF-α (**A**) and the total percentage of cytokine-producing CD8+ Trm lymphocytes and the percentage of CD8+ Tems producing any combination of IFN-γ, IL-2, or TNF-α (**B**) are presented as box-and-whisker plots (minimum to maximum), with individual values and the median indicated. Data were considered statistically significant at *p* < 0.05, as determined by one-way or two-way ANOVA followed by Tukey’s multiple comparison test (*: *p* < 0.05, **: *p* < 0.01).

Despite differences in tolerability, both vectors induced comparable adaptive immune responses, with similar frequencies of activated (CD69^+^) CD4^+^ and CD8^+^ T cells in the lungs (Figure 6). Likewise, the overall counts of effector memory T cells (Tems) did not differ substantially between the two vectors (Figure 7).

Functional analysis demonstrated that both vectors elicited robust cytokine-producing T-cell responses, including IFNγ^+^, IFNγ^+^TNFα^+^, and multifunctional IFNγ^+^TNFα^+^IL-2^+^ subsets, with comparable levels observed between NS124 and NS124_SS_CXCL10.

To further confirm that the comparable adaptive immune response was specifically associated with CXCL10 expression rather than with viral attenuation alone, we evaluated adaptive immunity following immunization with UV-inactivated NS124_SS_CXCL10. These experiments demonstrated that UV-inactivated NS124_SS_CXCL10 did not enhance adaptive immune responses, indicating that active CXCL10 expression contributes to the observed immunological effects. These data are presented in the Supplementary Materials (Figure S4).

### 3.5. CXCL10 Enhances Adaptive Immune Responses Following Parenteral Immunization

**Figure 8:**
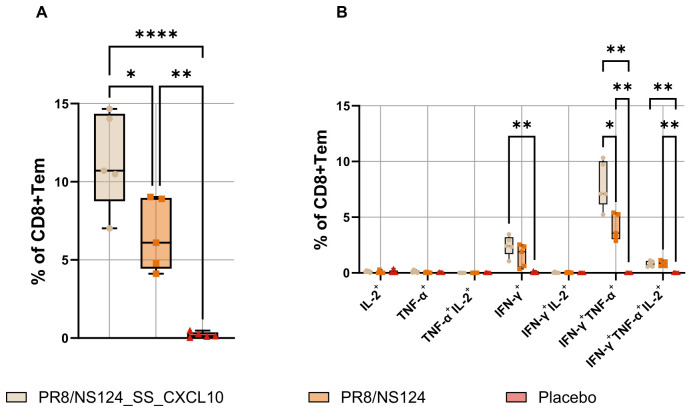
Antigen-specific CD8+ effector-memory T cells (Tem) response in the spleen. To evaluate splenic Tem responses, C57BL/6 mice were immunized intraperitoneally with the NS124_SS_CXCL10 (*n* = 5) or the NS124 (*n* = 5) strains at a dose of 7 log_10_ EID_50_ per mouse. The control group (*n* = 5) received an equivalent volume of PBS (500 μL per mouse). Spleens were collected at 10 d.p.im. Tem response in the spleen was evaluated by intracellular cytokine staining after 6 h of in vitro stimulation with the NP_366–374_ peptide. The total percentage of cytokine-producing CD8+ Tem cells (**A**) and the percentage of CD8+ Tem cells producing any combination of IFN-γ, IL-2, or TNF-α (**B**) are presented as box-and-whisker plots (minimum to maximum), with individual values and the median indicated. Data were considered statistically significant at *p* < 0.05, as determined by one-way or two-way ANOVA followed by Tukey’s multiple comparison test (*: *p* < 0.05, **: *p* < 0.01, ****: *p* < 0.0001).

**Figure 9:**
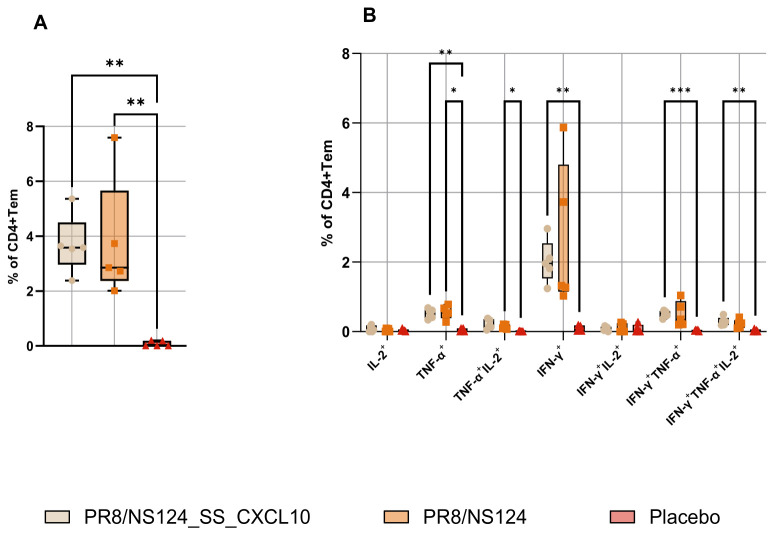
Antigen-specific CD4+ effector-memory T cells (Tem) response in the spleen. To evaluate splenic Tem responses, C57BL/6 mice were immunized intraperitoneally with the NS124_SS_CXCL10 (*n* = 5) or the NS124 (*n* = 5) strains at a dose of 7 log_10_ EID_50_ per mouse. The control group (*n* = 5) received an equivalent volume of PBS (500 μL per mouse). Spleens were collected at 10 d.p.im. Tem response in the spleen was assessed by intracellular cytokine staining after 24 h of in vitro stimulation with the A/PR8 wt virus. The total percentage of cytokine-producing CD4+ Tem cells (**A**) and the percentage of CD8+ Tem cells producing any combination of IFN-γ, IL-2, or TNF-α (**B**) are shown as box and whiskers plots (min and max with individual values and the median indicated). Data were considered statistically significant at *p* < 0.05, as determined by one-way or two-way ANOVA followed by Tukey’s multiple comparison test (*: *p* < 0.05, **: *p* < 0.01, ***: *p* < 0.001).

To evaluate the impact of CXCL10 expression on adaptive immune responses under non-replicating conditions, we employed a parenteral (intraperitoneal) immunization model in which both vectors exhibit minimal replication. Antigen-specific CD8^+^ and CD4^+^ effector-memory T cells producing IFN-γ, TNF-α, and IL-2 were quantified in the spleen 10 days post-immunization (Figure 8 and Figure 9).

Despite the lack of productive replication, mice immunized with the NS124_SS_CXCL10 vector developed significantly stronger CD8^+^ T-cell responses. The frequencies of IFN-γ^+^, IFN-γ^+^TNF-α^+^ and multifunctional IFN-γ^+^TNF-α^+^IL-2^+^ CD8^+^ T cells were markedly elevated compared to the placebo group (*p* < 0.01). In addition, the total pool of cytokine-producing CD8^+^ T cells was significantly increased in the CXCL10 group compared with the parental NS124 vector (*p* < 0.05) and the placebo group (*p* < 0.0001).

In the CD4^+^ T-cell population the NS124_SS_CXCL10 vector induced higher frequencies of TNF α^+^, IFN-γ^+^, IFN-γ^+^TNF-α^+^, and multifunctional IFN-γ^+^TNF-α^+^IL-2^+^CD4^+^ T cells compared with placebo group (*p* < 0.01). However, overall cytokine production was comparable between the CXCL10 and NS124 groups, with both vectors inducing significantly stronger responses than the placebo control (*p* < 0.01).

**Figure 10:**
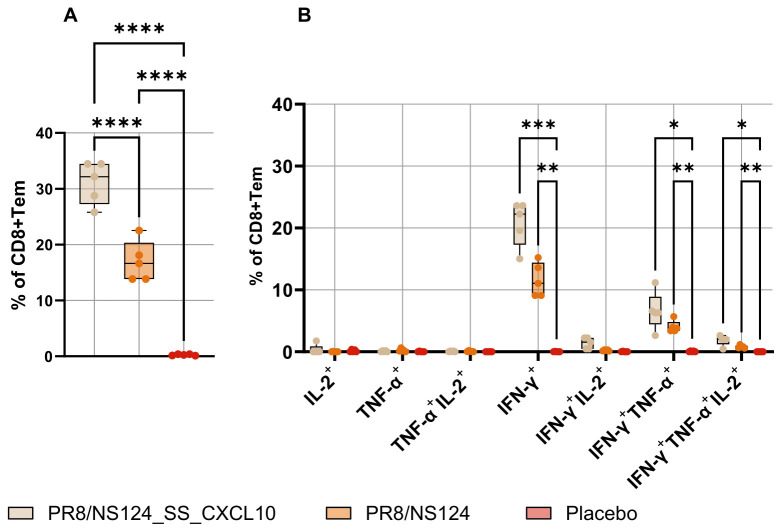
Antigen-specific CD8+ effector-memory T cells (Tem) response in the lungs. To evaluate mucosal T-cell responses after peritoneal immunization, animals were challenged with A/PR8 wt virus (3 log_10_ EID_50_ per mouse; *n* = 5) on day 21, and lungs were isolated on day 25. Tem response in the lungs was assessed using intracellular cytokine staining following 6 h of in vitro stimulation with the NP366–374 peptides. The total percentage of cytokine-producing CD8+ Tem cells (**A**) and the percentage of CD8+ Tem cells producing any combination of IFN-γ, IL-2, or TNF-α (**B**) are presented as box-and-whisker plots (minimum and maximum with individual values and the median indicated). Data were considered statistically significant at *p* < 0.05, as determined by one-way or two-way ANOVA followed by Tukey’s multiple comparison test (*: *p* < 0.05, **: *p* < 0.01, ***: *p* < 0.001, ****: *p* < 0.0001).

**Figure 11:**
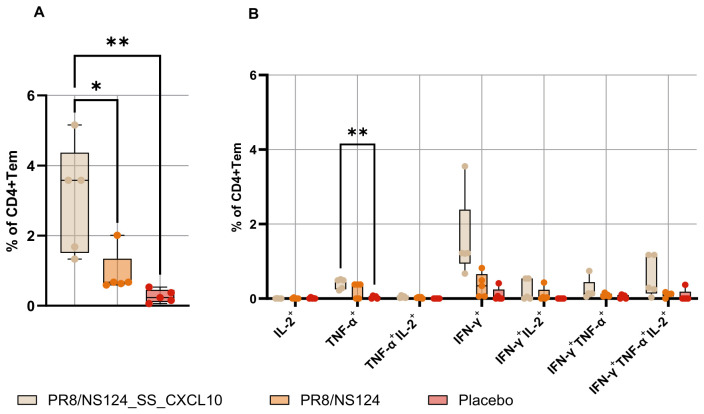
Antigen-specific CD4+ effector-memory T cells (Tem) response in the lungs. To evaluate mucosal T-cell responses after peritoneal immunization, animals were challenged with A/PR8 wt virus (3 log_10_ EID_50_ per mouse; *n* = 5) on day 21, and lungs were isolated on day 25. Tem response in the lungs was assessed via intracellular cytokine staining after 24 h of in vitro stimulation with A/PR8 wt virus. The total frequency of cytokine-producing CD4+ Tem cells (**A**) and the percentage of CD4+ Tem cells producing any combination of IFN-γ, IL-2, or TNF-α (**B**) are shown as box and whiskers plots (min and max with individual values and the median indicated). Data were considered statistically significant at *p* < 0.05, as determined by one-or two-way ANOVA followed by Tukey’s multiple comparison test (*: *p* < 0.05, **: *p* < 0.01).

To determine whether systemic immunization could also elicit mucosal memory, mice were infected intranasally with a very low dose of A/PR8 wt virus (3 log_10_ EID_50_ per mouse) on day 21, and lungs were collected on day 25 (Figure 10 and Figure 11). Following this low-dose challenge, NS124_SS_CXCL10 elicited the strongest CD8^+^ T-cell response in the lungs, with significantly elevated frequencies of IFN-γ^+^, IFN-γ^+^TNF-α^+^, and multifunctional IFN-γ^+^TNF-α^+^IL-2^+^ CD8^+^ cells compared to the placebo group (*p* < 0.05). Lung CD4^+^ T-cell responses showed a similar pattern, with significantly higher TNF-α^+^ CD4^+^ T-cell frequencies in the CXCL10 group (*p* < 0.01), whereas IL-2^+^ and IFN-γ^+^ subsets remained low and did not differ significantly. Despite its substantial attenuation in vivo, the CXCL10-expressing vector was nonetheless able to drive a significantly enhanced and functionally superior adaptive T-cell response.

### 3.6. CXCL10-Expressing Vector Confers Protection Despite an Attenuated Phenotype

To evaluate the protective efficacy of the CXCL10-expressing vector, C57BL/6 mice were intranasally immunized with 6 log_10_ EID_50_/30 μL per mouse. Twenty-one days after immunization, mice were challenged intranasally with A/Aichi/2/68 (H3N2) at a dose of 6 log_10_ EID_50_ per mouse in a total volume of 10 μL. Viral titers were measured in the lungs and nasal turbinates on days 3 and 5 post-infection.

**Figure 12:**
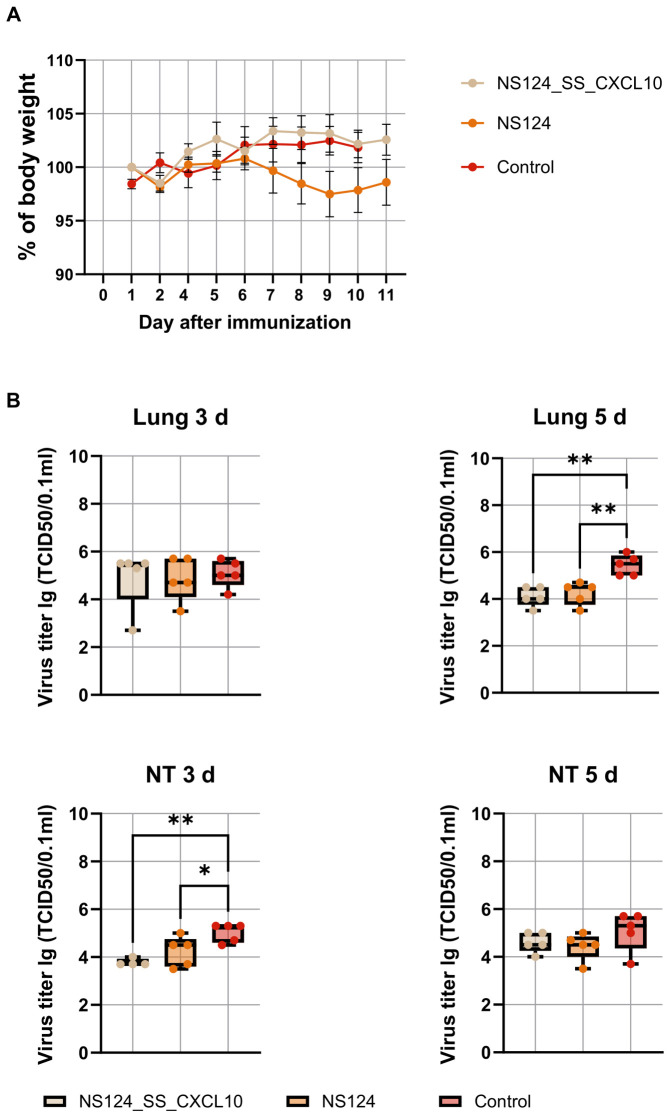
The protective efficacy of the CXCL10-expressing vector. C57BL/6 mice were immunized intranasally with 30 μL of 6 log10 EID_50_ per mouse (*n* = 10); control animals received PBS. On day 21 after immunization, mice were infected with 10 μL of the A/Aichi/2/68 (H3N2) strain. Body weight after immunization (**A**) and viral load in the nasal turbinates and lungs (**B**). Data were considered statistically significant at *p* < 0.05, as determined by one-way or two-way ANOVA followed by Tukey’s multiple comparison test (*: *p* < 0.05, **: *p* < 0.01).

Body weight was monitored for 11 days following immunization. Mice immunized with NS124 showed a transient decrease in body weight, whereas mice administered NS124_SS_CXCL10 maintained stable body weights comparable to controls (Figure 12).

On day 3 post-infection, viral titers in the lungs were comparable across all groups. By day 5 post-infection, both NS124_SS_CXCL10 and NS124 groups exhibited significantly lower viral titers compared to control animals. No significant differences were observed between the two immunized groups.

In the nasal turbinates, both immunized groups showed reduced viral titers compared to the non-immunized control group on day 3 post-challenge. No statistically significant differences were observed between the NS124_SS_CXCL10 and NS124 groups. On day 5 post-infection, viral titers were similar across all groups, with no statistically significant differences observed. Overall, NS124_SS_CXCL10 provided efficient viral clearance comparable to NS124 (Figure 12).

## 4. Discussion

In this study, we demonstrate that incorporation of CXCL10 into an NS1-truncated influenza A virus vector markedly enhances viral attenuation in vivo while preserving immunogenicity and protective efficacy. Despite efficient replication in embryonated chicken eggs and MDCK cells, the CXCL10-expressing vector exhibited strongly restricted replication in the respiratory tract of mice and did not induce weight loss even at high intranasal doses. Importantly, this improved safety without compromising immune function, as the vector retained its ability to induce robust adaptive immune responses and conferred protection against heterologous viral challenge comparable to the parental NS124 vector. These findings indicate that CXCL10 expression functionally uncouples attenuation from the loss of immunogenicity, providing a strategy to improve the safety of replication-competent influenza vectors without reducing their vaccine potential.

Mechanistically, our data suggest that attenuation of the CXCL10-expressing vector is driven not primarily by enhanced inflammatory cell recruitment, but rather by qualitative modulation of innate antiviral signaling. Although CXCL10 is classically associated with CXCR3-dependent chemotaxis [14,23], we observed no major differences in innate immune cell composition or early cytokine production between the CXCL10-expressing and parental vectors. Instead, the most prominent difference was increased IRF7 expression in the lungs, accompanied by comparable induction of other antiviral genes. In addition, mice immunized with the CXCL10-expressing vector exhibited significantly higher IFN-α levels in BAL at 8 h post-immunization, indicating an earlier activation of type I interferon responses. These observations suggest that CXCL10 does not simply amplify inflammation but instead shifts the host response toward a more interferon-centered antiviral state, potentially contributing to earlier control of viral replication. One possible explanation is that CXCL10 may enhance interferon-dependent signaling rather than directly induce antiviral gene expression. CXCL10 signaling through CXCR3 has been reported to modulate intracellular pathways, including activation of JAK/STAT and MAPK signaling cascades, which may increase cellular responsiveness to type I interferons and amplify local interferon feedback loops [14,24]. In this context, CXCL10 may potentiate interferon-driven antiviral programs, resulting in enhanced IRF7 expression without a corresponding increase in inflammatory cytokine production.

In parallel, CXCL10 may differentially modulate innate immune responses across the respiratory tract, thereby reinforcing this interferon-centered state in a spatially coordinated manner. The increased TLR8 expression and the tendency toward elevated TLR7 expression observed in the nasal turbinates suggest enhanced early viral sensing at the primary site of infection, potentially leading to more efficient initiation of antiviral signaling. In contrast, in the lungs, the dominant effect appears to be amplification of downstream interferon responses, reflected by increased IRF7 expression. Together, these effects may promote more efficient early detection of the virus in the upper airways, combined with rapid antiviral control in the lower respiratory tract, resulting in accelerated viral restriction without excessive inflammatory activation.

Importantly, CXCL10-mediated attenuation did not impair adaptive immune induction. Following intranasal immunization, the CXCL10-expressing vector elicited T-cell responses comparable to those induced by the parental NS124 vector despite its reduced replication. Under intraperitoneal conditions, where viral replication was minimal for both vectors, CXCL10 expression resulted in significantly enhanced antigen-specific CD8^+^ and CD4^+^ effector-memory responses. These findings indicate that CXCL10 enhances adaptive immune priming in a manner not solely dependent on viral replication, consistent with a role for locally expressed CXCL10 as an intrinsic immunomodulatory signal.

Despite its restricted replication phenotype, the CXCL10-expressing vector maintained protective efficacy in vivo. Immunized animals demonstrated efficient control of heterologous H3N2 challenge, with reduced viral loads in both the upper and lower respiratory tract. Viral titers in the nasal turbinates and lungs were comparable to those observed in animals immunized with the parental NS124 vector, indicating that attenuation of the CXCL10-expressing virus did not compromise its protective capacity. These findings suggest that extensive viral replication in the lungs is not required for effective protection when appropriate innate and adaptive immune responses are induced.

Taken together, these results establish CXCL10 expression as a strategy for rational improvement of influenza viral vectors. By enhancing attenuation while preserving or augmenting adaptive immunity, CXCL10 addresses a central limitation of live viral vector design. More broadly, these findings support the concept that targeted modulation of innate immune signaling—rather than simple amplification of inflammation—can be used to optimize the balance between safety and efficacy in replication-competent viral vaccines.

## Data Availability

The data presented in this study are available on reasonable request from the corresponding author. The data are not publicly available because they form part of ongoing research.

## Supplementary Materials

The following supporting information can be downloaded at https://www.mdpi.com/article/10.3390/pharmaceutics18060739/s1: Table S1. Primer and probe sequences used to assess of mouse gene expression; Figure S1. Schematic Representation of Experimental Designs; Figure S2. Early cytokine profile in the peritoneal cavity; Figure S3. Early innate immune cell recruitment; Figure S4. Antigen-specific CD8+ Trm and CD4+ Trm response in the lung.

## Abbreviations

The following abbreviations are used in this manuscript:

IAV	influenza A virus
ANOVA	analysis of variance
DCs	Dendritic cells
CXCR3	C-X-C motif receptor 3
CXCL10	high serum C-X-C motif chemokine ligand 10
NK	natural killer
NS	nonstructural protein
ECE	embryonated chicken eggs
EID_50_	50% embryonic infectious dose
TCID_50_	50% culture infectious dose
TMB	3,3′,5,5′-Tetramethylbenzidine
MDCK	Madin-Darby canine kidney cell
ELISA	enzyme-linked immunosorbent assay
PCR	polymerase chain reaction
SEM	standard error of the mean
SD	standard deviation
PBS	phosphate-buffered saline
