# Supplementary material for: A CXCL10-Expressing Influenza Vector Induces Robust Adaptive Immunity Despite Strong Attenuation
Source: Pharmaceutics. 2026 Jun 14;18(6):739. doi: 10.3390/pharmaceutics18060739 (PMC13306579; doi:10.3390/pharmaceutics18060739)
Supplement: Supplementary file 1 [file pharmaceutics-18-00739-s001.zip › pharmaceutics-4350565-supplementary.pdf]

pharmaceutics

MDPI

A CXCL10-Expressing Influenza Vector Induces Robust Adaptive Immunity Despite Strong Attenuation

Olga Ozhereleva *, Alina Mustafaeva, Anastasia Pulkina, Marina Plotnikova, Marina Shuklina, Anna-Polina Shurygina, Marina Stukova, Andrej Egorov

Supplementary

Figure S1. Schematic Representation of Experimental Designs

Experiment 1. Experimental Design for Assessment of Adaptive Immune Responses Following Intranasal Immunization at a dose of 6 log_{10} EID_{50}

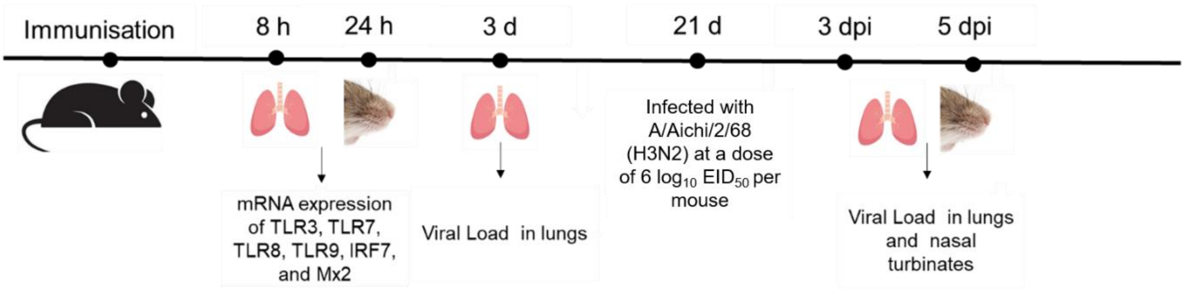

Experiment 2. Experimental Design for Assessment of Adaptive Immune Responses Following Intranasal Immunization at a dose of 7 log_{10} EID_{50}

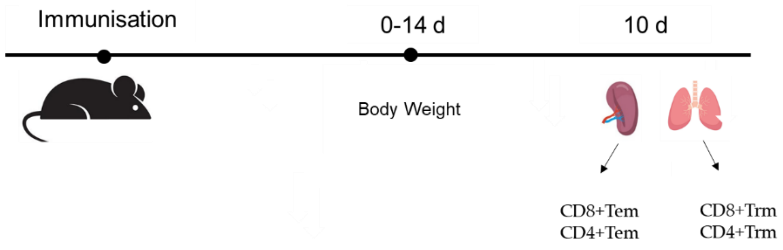

Experiment 3. Experimental Design for Assessment of Adaptive Immune Responses Following Intraperitoneal Immunization at a dose of 7 log_{10} EID_{50}

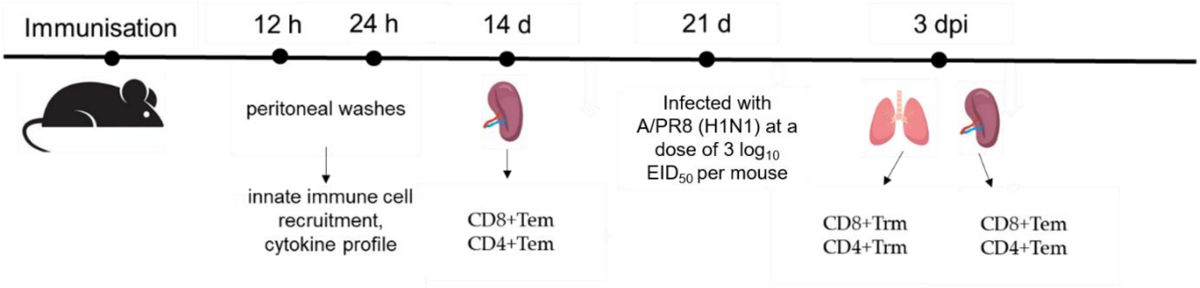

pharmaceutics

MDPI

Experiment 4. Experimental Design for Assessment of Adaptive Immune Responses Following Intranasal Immunization at a dose of 6 log_{10} EID_{50}

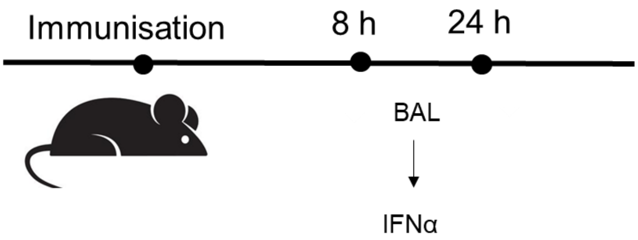

Table S1. Primer and probe sequences used to assess of mouse gene expression.

Gene	Primers/Probe	Sequence
OAS1ag	Forward	GATGTGCCGACGGTGGT
	Reverse	TGGACAGGAGTCAAACATGGC
	Probe	(Quasar705)-CACCTGGCATCAGACTCCGTGCTTCT-(BHQ2)
IRF7	Forward	CCTGGAAGCATTTCGGTCGGT
	Reverse	CTCTTCGCTCTCTTCGCTCA
	Probe	(HEX)-CCACCTAGTGGAGTTAACCTGCCA-(BHQ1)
TLR7	Forward	TCATGTGCCATGCTCAGTT
	Reverse	GGCAGATGTGTGGCTCTTAT
	Probe	(FAM)-TGTGCCTAGGAGACAACACAAGGC-(BHQ1)
RIG I	Forward	CTGTATAGCTTTGGCTGTCCT
	Reverse	CGCCTTTAATCCCAACACTTG
	Probe	(FAM)-ACTCAGAAATCCGCCTTCCTCTGC-(BHQ1)
Mda5	Forward	GGTTCAGGCTTGCTTCTTCT
	Reverse	ACTCCCTTCATCATAAGAGATGATTAG
	Probe	(FAM)-TCTTCTGCAAACACAGTACCATCCTGG-(BHQ1)
TLR3	Forward	TGGTCACCAACTGGCTATTAAA
	Reverse	CCATCTATCACTGTGGCTCTTC
	Probe	(FAM)-ACCCATGCCTGAGTAGTCTTCTCTGA-(BHQ1)
TLR8	Forward	AGGCAGCTTATTATGCCTACTT
	Reverse	TGTATCAACTTCACCAGCATCT
	Probe	(FAM)-TCAATCCCTAAGAACATTTGCCACTGT-(BHQ1)
Mx2	Forward	GTTAAGCTGGCTCTGTCCTT
	Reverse	AGCATAAAGGCTGGTGGTATAG
	Probe	HEX-TTGTGATTCAGGGACAGAAGGGCT-BHQ1
IL6	Forward	TGATGGATGCTACCAAACTGGA
	Reverse	CTGAAGGACTCTGGCTTTGTCT

	Probe	(ROX)-CTTCTGGAGTACCATAGCTACCTGGAGTA-(BHQ2)
HPRT1	Forward	GAAGCTCTCGATTTCCTATCAGT
	Reverse	CAACGATTTACTGAAAGTGGGAAA
	Probe	(HEX)-ACATGTTTCAGCAGTGTTGGCTGT-(BHQ1)
Rplp0	Forward	CAAAGGAAGAGTCGGAGGAATC
	Reverse	CTTTCTCAAATTAAGCAGGCTGAC
	Probe	(ROX)-TCTTCGACTAATCCCGCCAAAGCA-(BHQ2)
Ubc	Forward	CCCAGTGTTACCACCAAGAAG
	Reverse	CCCATCACACCCAAGAACAA
	Probe	(Cy5)-AGACAGACGTACCTTCCTCACCACA-(BHQ2)
GAPDH	Forward	AATGGTGAAGGTCGGTGTG
	Reverse	ACAAGCTTCCCATTCTCGG
	Probe	(HEX)-TTGACTGTGCCGTTGAATTTGCCG-(BHQ1)

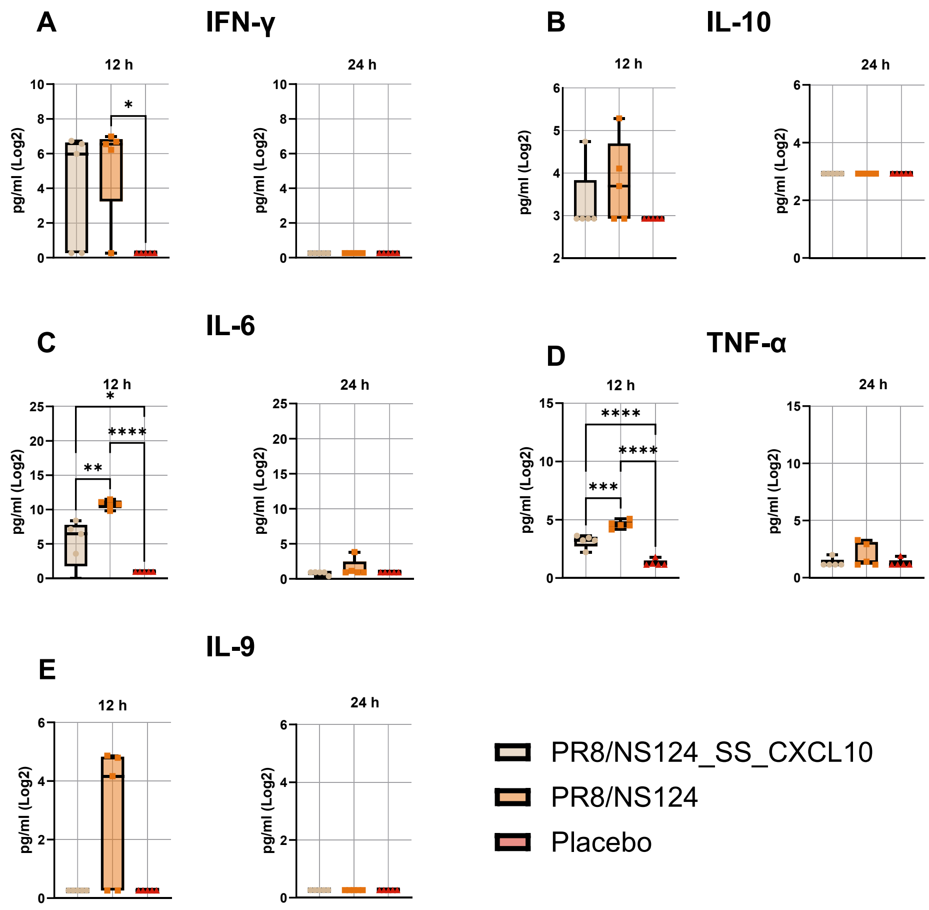

Figure S2. Early cytokine profile in the peritoneal cavity. To measure cytokine concentrations C57BL/6 mice were immunized intraperitoneally with the NS124_SS_CXCL10 or the NS124 (empty vector) strains at a dose of 7 $\log_{10}$

EID50 per mouse. The control group received PBS in the corresponding volume (500 µl per mouse). Peritoneal washes were collected 12 and 24 hours after immunization. Cytokine concentration was measured using the LEGENDplex multiplex system (Biolegend, USA) following the manufacturer's instructions. Concentrations of IFN-γ, (A), IL-6 (B), TNF-α (C), IL-10 (D) and IL-9 (E) are presented as box-and-whisker plots (minimum to maximum, with individual values and the median indicated). Differences were considered statistically significant at $p < 0.05$, as determined by one-way ANOVA followed by Tukey's multiple comparison test (*: $p < 0.05$, **: $p < 0.01$, ***: $p < 0.001$, ****: $p < 0.0001$).

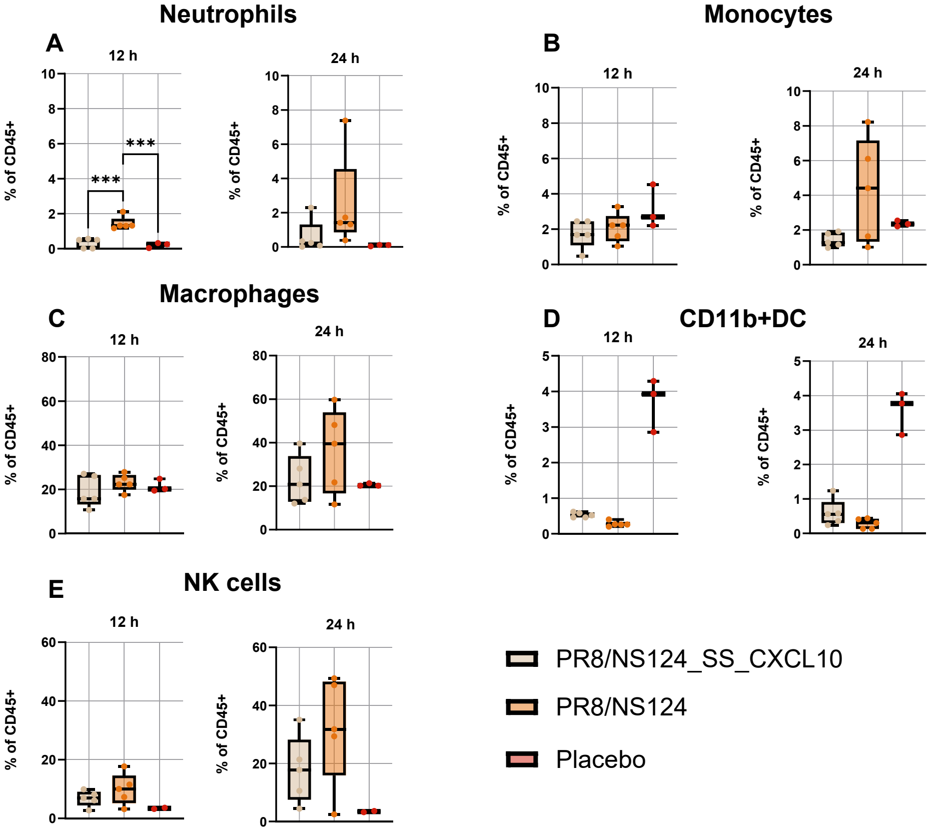

Figure S3. Early innate immune cell recruitment. To measure innate immune cell recruitment C57BL/6 mice were immunized intraperitoneally with the NS124_SS_CXCL10 or the NS124 (empty vector) strains at a dose of 7 $\log_{10}$ EID50 per mouse. The control group received PBS in the corresponding volume (500 µl per mouse). Peritoneal washes were collected 12 and 24 hours after immunization. Innate immune response in the peritoneal cavity was assessed at 12- and 24-hours p.imm. The proportion of neutrophils among the parent population (CD45+ live cells) (A), the percentage of monocytes in the grandparent population (CD45+ live cells) (B), the percentage of macrophages in the grandparent population (CD45+ live cells) (C), and the percentage of dendritic cells in the grandparent population (CD45+ live cells) (D) is presented as box-and-whisker plots (minimum to maximum). Differences were considered statistically significant at $p < 0.05$, as determined by one-way ANOVA followed by Tukey's multiple comparison test (***: $p < 0.001$).

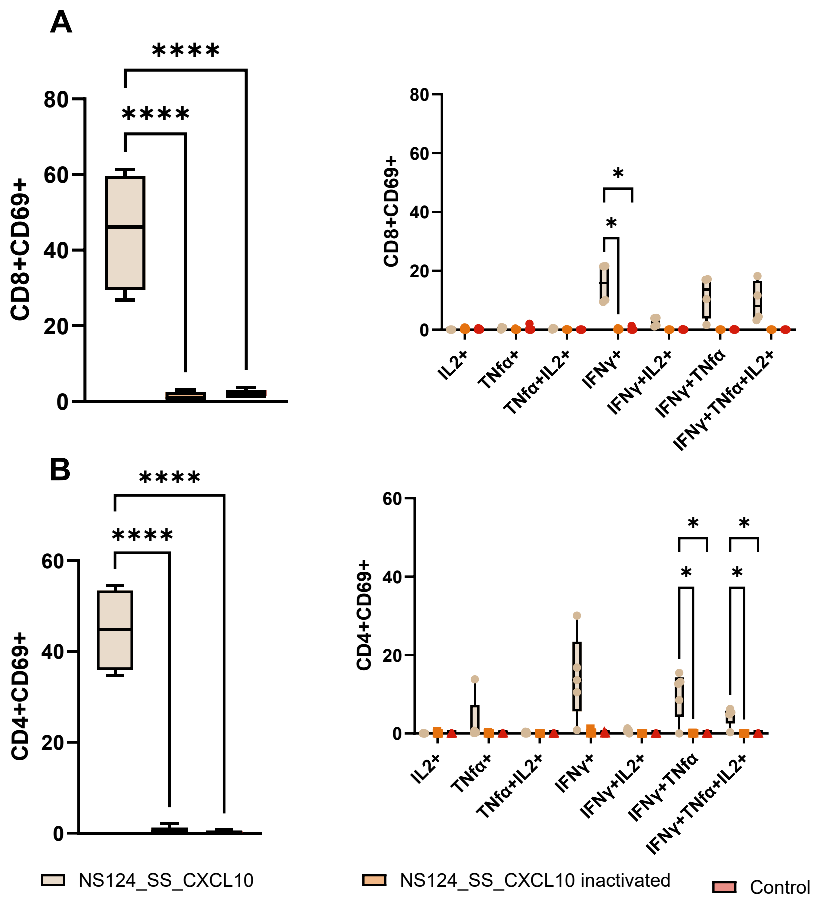

Figure S4. Antigen-specific CD8+Trm and CD4+Trm response in the lung. To measure Trm response in the spleen C57BL/6 mice were immunized intranasally with the NS124_SS_CXCL10 or the NS124_Ss_CXCL10 inactivated strains at a dose of 6 $\log_{10}$ EID_{50} per mouse. The control group received PBS in the corresponding volume (30 µl per mouse). lung were collected at 10 d.p.im. Trm response in the lung was evaluated by intracellular cytokine staining after 6 h of in vitro stimulation with the NP366–374 peptide. The total percentage of cytokine-producing CD8+ Trm lymphocytes and the percentage of CD8+ Tem producing any combination of IFN-γ, IL-2, or TNF-α (A) and the total percentage of cytokine-producing CD4+ Trm lymphocytes and the percentage of CD8+ Tem producing any combination of IFN-γ, IL-2, or TNF-α (B) are presented as box-and-whisker plots (minimum to maximum), with individual values and the median indicated. Data were considered statistically significant at $p < 0.05$, as determined by one or 2-way ANOVA followed by Tukey's multiple comparison test (*: $p < 0.05$, **: $p < 0.01$, ***: $p < 0.001$, ****: $p < 0.0001$).
